# Focal adhesion kinase activation limits efficacy of Dasatinib in c‐Myc driven hepatocellular carcinoma

**DOI:** 10.1002/cam4.1777

**Published:** 2018-10-28

**Authors:** Xianqiong Liu, Xinhua Song, Jie Zhang, Zhong Xu, Li Che, Yu Qiao, Yunuen Ortiz Pedraza, Antonio Cigliano, Rosa M. Pascale, Diego F. Calvisi, Yanju Liu, Xin Chen

**Affiliations:** ^1^ School of Pharmacy Hubei University of Chinese Medicine Wuhan Hubei China; ^2^ Department of Bioengineering and Therapeutic Sciences and Liver Center University of California San Francisco California; ^3^ Beijing Advanced Innovation Center for Food Nutrition and Human Health College of Food Science and Nutritional Engineering China Agricultural University Beijing China; ^4^ Department of Thoracic Oncology II Key Laboratory of Carcinogenesis and Translational Research (Ministry of Education) Peking University Cancer Hospital & Institute Beijing China; ^5^ Department of Gastroenterology Guizhou Provincial People's Hospital The Affiliated People's Hospital of Guizhou Medical University Guiyang Guizhou China; ^6^ Department of Oncology Beijing Hospital National Center of Gerontology Beijing China; ^7^ Department of Health Science Universidad Autonoma Metropolitana‐Itapalapa Mexico City Mexico; ^8^ Institute of Pathology University of Greifswald Greifswald Germany; ^9^ Department of Clinical and Experimental Medicine University of Sassari Sassari Italy

**Keywords:** c‐Myc, Dasatinib, focal adhesion kinase, Lyn, Src

## Abstract

Hepatocellular carcinoma (HCC) is a deadly malignancy with limited treatment options. Recently, it was found that Dasatinib treatment led to synthetic lethality in c‐Myc high‐expressing human cancer cells due to inhibition of p‐Lyn. Overexpression of c‐Myc is frequently seen in human HCC. We investigated the sensitivity to Dasatinib in vitro using HCC cell lines and in vivo using c‐Myc mouse HCC model. We found that HCC cell line responsiveness to Dasatinib varied significantly. However, there was no correlation between c‐Myc expression and IC_50_ to Dasatinib. In c‐Myc‐induced HCC in mice, tumors continued to grow despite Dasatinib treatment, although the eventual tumor burden was lower in Dasatinib treatment cohort. Molecular analyses revealed that Dasatinib was effective in inhibiting p‐Src, but not p‐Lyn, in HCC. Importantly, we found that in HCC cell lines as well as c‐Myc mouse HCC, Dasatinib treatment induced up regulation of activated/phosphorylated (p)‐focal adhesion kinase(FAK). Concomitant treatment of HCC cell lines with Dasatinib and FAK inhibitor prevented Dasatinib‐induced FAK activation, leading to stronger growth restraint. Altogether, our results suggest that Dasatinib may have limited efficacy as single agent for HCC treatment. Combined treatment with Dasatinib with FAK inhibitor might represent a novel therapeutic approach against HCC.

## 1 INTRODUCTION

Hepatocellular carcinoma (HCC) is the fifth most common cancer and second leading cause of cancer deaths in the world.^1^, ^2^ Importantly, the incidence rate for HCC has been increasing during the past two decades.^3^, ^4^ Development of HCC is frequently associated with hepatitis B or C infection, alcohol abuse, as well as fatty liver disease. However, the precise mechanisms leading to HCC development remain poorly defined. HCC does not show specific symptoms at the early stage of the disease, and most patients are diagnosed with advanced stage HCC, which prevent them from receiving curative liver resection. Currently, treatment with Sorafenib or Regorafenib multi‐kinase inhibitors is the first‐line therapy for patients with advanced HCC. However, both drugs have poor efficacy and only prolong the survival for few months.^5^, ^6^ Thus, there is a great need to develop new therapeutic strategies against HCC.

c‐Myc is a well‐characterized oncogene in multiple tumor types.^7^ c‐Myc is a master regulator of transcription, contributing to tumor development via modulation of a large number of target genes.^8^, ^9^ In human HCC, c‐Myc has been shown to be frequently amplified and/or over expressed,^10^, ^11^ and its elevated levels have been found to be associated with poor survival.^10^ Importantly, when c‐Myc is over expressed in the mouse liver, it leads to HCC development.^12^, ^13^ Altogether, this body of evidence indicates that c‐Myc is a driver oncogene for HCC. Despite the key role of c‐Myc in tumorigenesis, its suppression by direct inhibition is difficult to achieve.^14^ In recent years, significant efforts have been devoted to screen for genetic or metabolic events that may lead to synthetic lethality for c‐Myc.^15^ In a recent study, using chemical‐genetic interaction mapping technology, it was uncovered that c‐Myc overexpressing cells are highly sensitive to Dasatinib.^16^ Further mechanistic studies revealed that cancer cells harboring elevated levels of c‐Myc require the Lyn kinase for survival and that Dasatinib inhibits c‐Myc overexpressing cells by negatively regulating the Lyn protein function.^16^

Dasatinib is a highly potent, ATP‐competitive, and orally active dual Src/Abl kinase inhibitor possessing anti‐proliferative activity against multiple tumor types. Currently, Dasatinib is approved for treatment of chronic myeloid leukemia patients who are resistant or cannot tolerate Imatinib. It has also been successfully used for treating Philadelphia (+) acute lymphoblastic leukemia and to robustly inhibit the growth of solid tumor cells, including breast, prostate, colon, and lung cancer.^17^ In human liver cancer, a subset of HCC cell lines was found to be highly sensitive to Dasatinib administration.^12^, ^18^ However, the precise mechanisms whereby Dasatinib induces HCC growth inhibition remain controversial. Whether c‐Myc is a valid biomarker for sensitivity to Dasatinib in HCC is not known.

In this study, we evaluated whether c‐Myc overexpressing HCC cells are sensitive to Dasatinib‐based therapy in vitro and in vivo. We found that c‐Myc and p‐Lyn expression did not predict Dasatinib responsiveness in HCC cell lines. Similarly, Dasatinib showed limited antineoplastic efficacy in c‐Myc mice. Importantly, we found that Dasatinib treatment induces activation of Focal Adhesion Kinase (FAK), which might limit its therapeutic efficacy. Indeed, concomitant treatment of HCC cells with Dasatinib and FAK inhibitor induced higher growth constraint than the single drug alone. Altogether, the present data suggest that Dasatinib might have limited efficacy as single agent for HCC treatment. Therapeutic strategies consisting of Dasatinib and a FAK inhibitor might be highly beneficial for the treatment of human HCC patients.

## 2 MATERIALS AND METHODS

### 2.1 Reagents and chemicals

The plasmids used for the study, including pT3‐EF1α‐c‐Myc (Addgene #92046), and pCMV/sleeping beauty transposase (pCMV/SB; Addgene # 24551), were described previously^13^, ^19^ and were deposited at Addgene (Cambridge, MA, USA). For in vivo studies, plasmids were purified using the Endotoxin Free Maxi Prep Kit (Sigma‐Aldrich, St. Louis, MO, USA). Dasatinib was purchased from LC Laboratories (Woburn, MA, USA). PND‐1186 was purchased from Selleck Chemicals (Houston, TX, USA). For in vitro use, Dasatinib was dissolved in DMSO (ThermoFisher Scientific, Waltham, MA, USA) at a concentration of 50 mmol/L, while PND‐1186 was dissolved in DMSO at a concentration of 10 mmol/L. Table S1 for the detailed product information.

### 2.2 Hydrodynamic tail vein injection and mouse treatment

Wild‐type FVB/N mice were obtained from Jackson Laboratory (Bar Harbor, ME). To induce HCC formation in the liver, we performed hydrodynamic injections as described previously.^20^ Specifically, 20 μg pT3‐EF1α‐c‐Myc and 0.8 μg pCMV/SB were diluted in 2 mL saline for each mouse. Plasmid mixture was injected into the lateral tail vein of 6‐ to 7‐week‐old female FVB/N mice within 7 seconds. After mice developed palpable abdominal mass, mice were grouped randomly into two cohorts and treated with either oral administration of Dasatinib (25 mg/kg/d) or vehicle (1% DMSO) for 6 days per week. The animal studies followed the protocol which was approved by the Committee for Animal Research at the University of California, San Francisco.

### 2.3 Histology and immunohistochemistry

Mouse liver samples were fixed in zinc formal‐fixx (ThermoFisher Scientific) and embedded in paraffin. Hematoxylin (ThermoFisher Scientific) and eosin (ThermoFisher Scientific; H&E) stained slides were used for histology evaluation. Immunohistochemistry was performed as described previously.^13^ In brief, slides were deparaffinized in xylene, rehydrated through a graded alcohol series and finally, rinsed in PBS. Antigen unmasking was performed via boiling the slides in 0.01 mol/L citrate buffer (pH 6.0) for 10 minutes. The slides were first incubated with 5% goat serum and Avidin‐Biotin Blocking Kit (Vector Laboratories, Burlingame, CA, USA); and, subsequently, with primary antibodies at 4°C for 16 hours. After washing, slides were incubated with 3% hydrogen peroxide for 10 minutes followed by incubating with biotin conjugated secondary antibody for additional 30 minutes at room temperature. Vectastain ABC Elite Kit (Vector Laboratories) was used to visualize the signal. Finally, slides were counterstained with hematoxylin. Slides incubated without primary antibodies were used as negative control. Images were taken using bright‐field microscope with Digital color camera (Model# DFC295; Leica, San Francisco, CA, USA). Detailed information for the reagents could be found in Table S1 and antibody information in Table S2.

### 2.4 Protein extraction and western blotting

Protein was extracted from liver tissues or cell pellets using Mammalian Protein Extraction Reagent (ThermoFisher Scientific) containing the Complete Protease Inhibitor Cocktail (Roche Molecular Biochemicals, Indianapolis, IN, USA). Protein concentration was quantified using the Protein Assay Kit (Bio‐Rad, Hercules, CA, USA). For each analysis, we loaded 30 μg aliquots of denatured lysates onto SDS‐PAGE gel (Bio‐Rad), and transferred the proteins onto nitrocellulose membranes (Bio‐Rad) for 3‐10 minutes using trans‐Blot Turbo System (Bio‐Rad). Membranes were blocked in 5% nonfat dry milk in TBST 1 hour and then incubated with primary antibodies (Table S1) in TBST with 5% BSA (#A3294, Sigma‐Aldrich) at 4°C cold room overnight. After washing, blots were incubated with horseradish peroxidase‐secondary antibody (Jackson Immuno Research Laboratories Inc., West Grove, PA, USA) at 1:10 000 for 1 hour and then visualized by the Super Signal West Femto (Pierce Chemical Co., New York, NY, USA) reagent and then were exposed to X‐ray films. Image‐J software (http://imagej.nih.gov/ij/) was used for densitometric analysis of the blots. Detailed information for the reagents could be found in Table S1 and antibody information in Table S2.

### 2.5 In vitro experiments

The following human HCC cell lines were used for the in vitro studies: Focus, Hep40, HLE, HLF, MHCC97H, Huh7, PLC/PRF/5, SK‐HEP1, SNU‐398, SNU‐449, and SNU‐475. In addition, the mouse HCC3‐4, HCC4‐4 cell lines, derived from HCC developed in c‐Myc transgenic mice,^21^ were used. Cell lines were cultured in Dulbecco's modified Eagle's medium supplemented with 10% fetal bovine serum (FBS; Gibco, Grand Island, NY, USA), 100 U/mL penicillin and 100 μg/mL streptomycin (Sigma‐Aldrich). All experiments were repeated at least three times in triplicate. Detailed information for the reagents could be found in Table S1 and cell line information in Table S3.

Cell viability assays were performed using crystal violet (Sigma‐Aldrich) staining. In brief, cells were seeded in 24‐well plates and treated in triplicate with Drugs at the concentrations indicated in the manuscript for 48 hours. Cells were stained with crystal violet. After washing, stained cells were incubated with lysis solution for 20‐30 minutes. Diluted lysate solutions were added to 96‐well plates, and OD was measured at 590 nm with an ELx808 Absorbance Microplate Reader (BioTek, Winooski, VT, USA). Cell proliferation rate was determined using the BrdU Cell Proliferation Assay Kit (Cell Signaling Technology, Danvers, MA, USA); whereas cell apoptosis rate was measured using the Cell Death Detection Elisa Plus Kit (Roche Molecular Biochemicals) following the manufacturers’ instructions. All cell line experiments were repeated at least three times in triplicate. Detailed information for the reagents could be found in Table S1.

### 2.6 Statistical analysis

Data analysis was performed using Prism 6 Software (GraphPad, San Diego, CA, USA). All data are presented as Means ± SD. Mann‐Whitney *U* tests were applied. *P* values <0.05 were considered statistically significant.

## 3 RESULTS

### 3.1 Lack of correlation between c‐Myc expression and Dasatinib sensitivity in a panel of HCC cell lines

**Figure 1:**
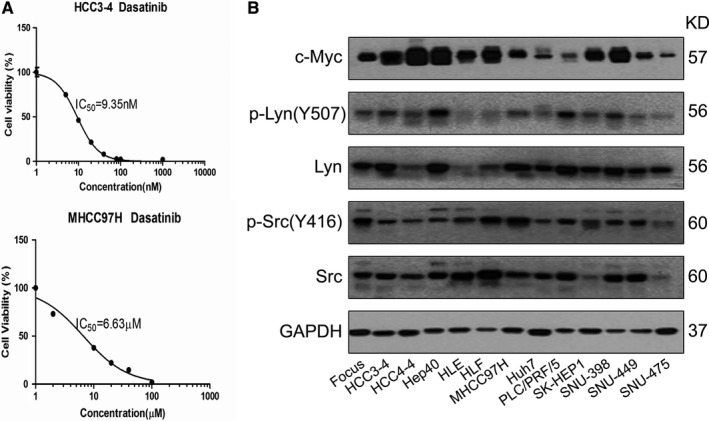
Lack of correlation between c‐Myc expression and Dasatinib sensitivity in a panel of HCC cell lines. A, Representative IC_50_ against Dasatinib in two HCC cell lines (HCC3‐4 and MHCC97H cells). B, Expression of c‐Myc, p‐Lyn and p‐Srcin a panel of 11 human HCC cell lines (Focus, Hep40, HLE, HLF, MHCC97H, Huh7, PLC/PRF/5, SK‐HEP1, SNU‐398, SNU‐449, SNU‐475) and 2 mouse HCC cell lines (HCC3‐4 and HCC4‐4)

We determined the IC_50_ against Dasatinib in a panel of 11 human HCC cell lines (Focus, Hep40, HLE, HLF, MHCC97H, Huh7, PLC/PRF/5, SK‐HEP1, SNU‐398, SNU‐449, and SNU‐475) and two mouse HCC cell lines derived from liver specific c‐Myc transgenic mice (HCC3‐4 and HCC4‐4).^21^ Consistent with a previous report,^12^ we found that Dasatinib showed a highly heterogeneous anti‐growth activity in HCC cells, with IC_50_ ranging from ~10 nmol/L to ~10 μmol/L (Table 1, Figure 1A and Figure S1). Next, we measured the levels of c‐Myc, p‐Lyn, and p‐Src in the same panel of cell lines using Western blotting (Figure 1B). Of note, we found that these proteins exhibit variable expression levels in HCC cells (Table 1 and Figure 1B). Subsequently, we determined whether there was any correlation between Dasatinib IC_50_ values and c‐Myc, p‐Lyn, and p‐Src levels in HCC cell lines. We found that there were cell lines with high c‐Myc expression and low IC_50_ against Dasatinib, such as HCC3‐4 cells; but also cell lines with high c‐Myc expression but high IC_50_ against Dasatinib, such as HLF cells (Table 1). Using statistical analysis, we found that there was no correlation between c‐Myc levels and Dasatinib IC_50_ (*R* = 0.07, *P *=* *0.82; Figure S2A). Furthermore, no correlation between p‐Lyn expression and Dasatinib IC_50_ (*R* = −0.17, *P *=* *0.58; Figure S2B), or p‐Src levels and Dasatinib IC_50_ (*R* = 0.25, *P *=* *0.41; Figure S2C) was detected. We also investigated the correlation between c‐Myc, p‐Lyn, and p‐Src expression in HCC cells. The analysis revealed a moderate positive correlation between c‐Myc and p‐Lyn levels (*R* = 0.5, *P *=* *0.08; Figure S2D), but no correlation between c‐Myc and p‐Src (*R* = −0.41, *P *=* *0.16; Figure S2E), or p‐Lyn and p‐Src levels (*R* = −0.41, *P *=* *0.17; Figure S2F).

**Table 1:** IC_50_ against Dasatinib in a panel of mouse and human HCC cell lines

Cell lines	IC_50_ (nmol/L)	p53 status	Cell line type	p‐Lyn/Lyn	p‐Src/Src	c‐Myc/GAPDH
Focus	4083	Absent	Human HCC	0.95	1.27	1.18
HCC3‐4	9	Wide type	Mouse c‐Myc HCC	0.78	0.54	2.42
HCC4‐4	18	Wide type	Mouse c‐Myc HCC	1.23	0.53	2.97
Hep40	872	Mutation	Human HCC	1.05	0.45	4.66
HLE	6977	Mutation	Human HCC	0.86	0.48	1.95
HLF	5930	Mutation	Human HCC	0.52	0.74	3.85
MHCC97H	6630	Mutation	Human HCC	0.5	0.94	1.46
Huh7	1078	Mutation	Human HCC	0.68	0.48	0.7
PLC/PRF/5	1722	Mutation	Human HCC	0.66	0.56	0.99
SK‐HEP1	3937	Wide type	Human HCC	0.67	1.92	1.76
SNU398	9800	Mutation	Human HCC	0.85	0.6	4.18
SNU449	17	Mutation	Human HCC	0.51	0.6	1.27
SNU475	500	Mutation	Human HCC	0.37	1.86	0.38

Previous studies have suggested that p53 mediated autophagy might contribute to Dasatinib responsiveness in cancer cells.^22^ We therefore investigated whether p53 status was associated Dasatinib sensitivities in HCC cell lines. We did not detect any correlation between p53 status and Dasatinib IC_50_ (Table 1).

In summary, we show that Dasatinib displays a highly variable antigrowth efficacy against HCC cells, which is not associated with baseline c‐Myc, p‐Src, p‐Lyn expression levels or p53 status.

### 3.2 Dasatinib treatment triggers FAK activation in HCC cell lines

**Figure 2:**
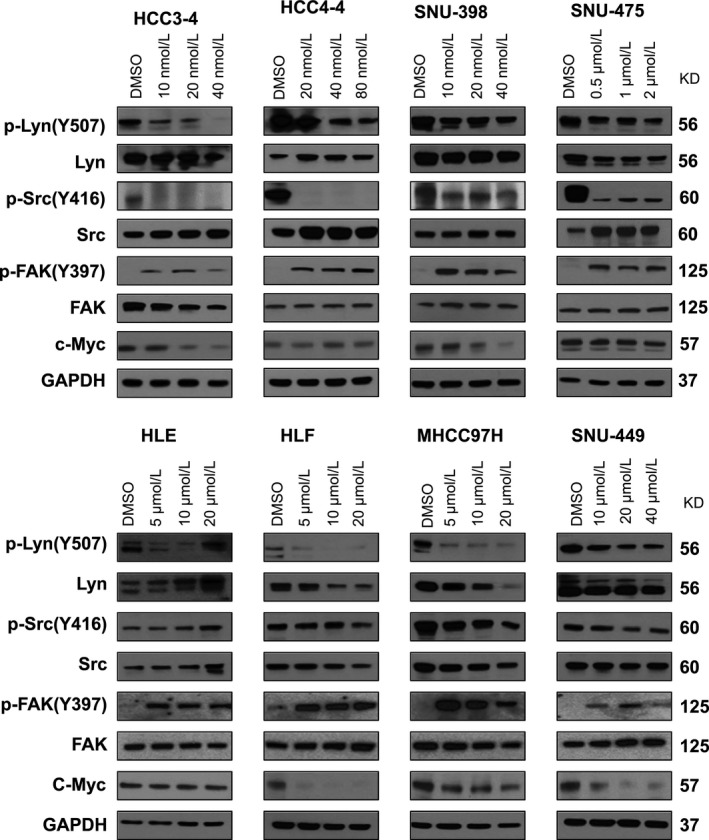
Molecular analysis of Dasatinib treated HCC cell lines. HCC3‐4, HCC4‐4, SNU‐398, SNU‐475, HLE, HLF MHCC97H, and SNU‐449 cells were treated with Dasatinib at IC50 concentration, and expression of c‐Myc, p‐Lyn, Lyn, p‐Src, Src, p‐FAK and FAK was analyzed using Western blotting. GAPDH was used as loading control

Next, we investigated the biochemical effects of Dasatinib in HCC cell lines. For this purpose, the panel of HCC cell lines was treated with Dasatinib at concentration around IC_50_. We found that, at the effective dose, Dasatinib was able to suppress p‐Src and p‐Lyn levels in most of the HCC cell lines examined (Figure 2). Dasatinib treatment led to the decreased c‐Myc expression levels in some HCC cell lines tested. However this phenotype did not appear to be correlated with sensitivities to Dasanitib (Figure 2). As FAK is considered to be a main target of Dasatinib,^23^ we analyzed whether Dasatinib was able to inhibit FAK activity in HCC cell lines. Surprisingly, we found that Dasatinib induced an up‐regulation of activated/phosphorylated (p)‐FAK in all the cell lines tested. Because Dasatinib has been shown to regulate additional targets, such as Stat3,^24^ NF‐κB,^25^ Ras/MAPK and AKT/mTOR^26^ pathways, we analyzed the expression of these targets in a panel of 4 mouse and human HCC cell lines (Figure S3). We found that at high dose, Dasatinib was able to inhibit p‐ERK and p‐RPS6 expression.

Altogether, our study suggests that Dasatinib can inhibit p‐Src and p‐Lyn expression in HCC cell lines in culture. However, Dasatinib paradoxically triggers activation of FAK in HCC cells, which may limit it therapeutic efficacy.

### 3.3 Limited efficacy of Dasatinib against c‐Myc driven hepatocarcinogenesis in mice

Since in vitro cell culture experiments do not necessarily recapitulate in vivo studies, we investigated the activity of Dasatinib against c‐Myc induced HCC in mice. Previously, we established a c‐Myc driven murine HCC model by hydrodynamically injecting c‐Myc into the mouse liver (c‐Myc mice).^13^, ^27^ These mice were found to develop HCC lesions starting ~3 weeks post hydrodynamic injection, and mice had to be euthanized due to high liver tumor burden by 7 weeks post injection.^13^, ^27^

**Figure 3:**
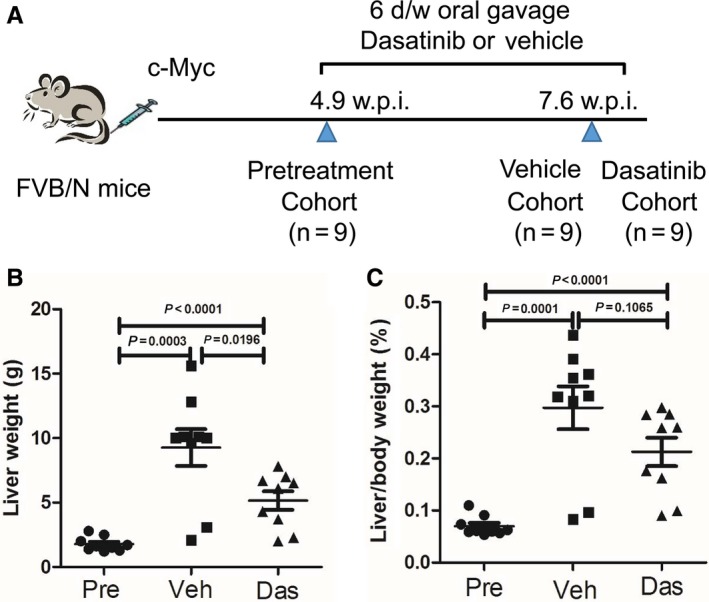
Therapeutic efficacy of Dasatinib for the treatment of HCC developed in c‐Myc mice. A, Study design. B, Liver weight comparison in pre‐treated (n = 9), vehicle (n = 9), and Dasatinib treated (n = 9) FVB/N mice. (C) Liver/body weight comparison in pre‐treated(n = 9), vehicle(n = 9), and Dasatinib treated (n = 9) FVB/N mice. Data are presented as mean ± SD; and *P*‐value was calculated using Mann‐Whitney *U* test. Each dot represents one value for one mouse. Das, Dasatinib; Pre, Pre‐treatment; Veh, Vehicle

Although Dasatinib has been previously used in preclinical studies, it was never utilized in FVB/N mice. Thus, we first tested the maximum tolerated dose of Dasatinib in FVB/N mice. For this purpose, FVB/N mice were subjected to the administration of the following doses of Dasatinib for 5 consecutive days: 15, 25, or 50 mg/kg/d. We found that at 50 mg/kg/d, Dasatinib was highly toxic, while at 25 mg/kg/d Dasatinib appeared to be well‐tolerated by FVB/N mice. Therefore, the dose of 25 mg/kg/d of Dasatinib was selected for the subsequent experiments. We hydrodynamically injected c‐Myc into the mouse liver (n = 27). Mice were aged until 4.9 weeks post injection when mice had moderate liver burden, and a cohort of 9 mice was harvested as “Pre‐treatment mice” (average liver weight ~ 2 g; Figure 3A). The remaining mice were randomly divided into two distinct cohorts and were treated with either vehicle (n = 9) or 25 mg/kg/d Dasatinib (n = 9) for 3 weeks (Figure 3A). Based on abdominal palpation, we realized that liver tumors continued to grow in both cohorts. Upon dissection, we found that all mice developed liver tumors. The medium liver weight for the vehicle treatment cohort was ~9 g, whereas was ~5 g in the Dasatinib treatment cohort (Figure 3B). The difference between the two cohorts was statistically significant (*P *<* *0.05; Figure 3B). Importantly, both cohorts displayed higher liver weight than that of the pre‐treatment group, indicating that tumor progression occurred in these mice (Figure 3B). Similar conclusions were reached after assessing liver/body weight ratio (Figure 3C).

**Figure 4:**
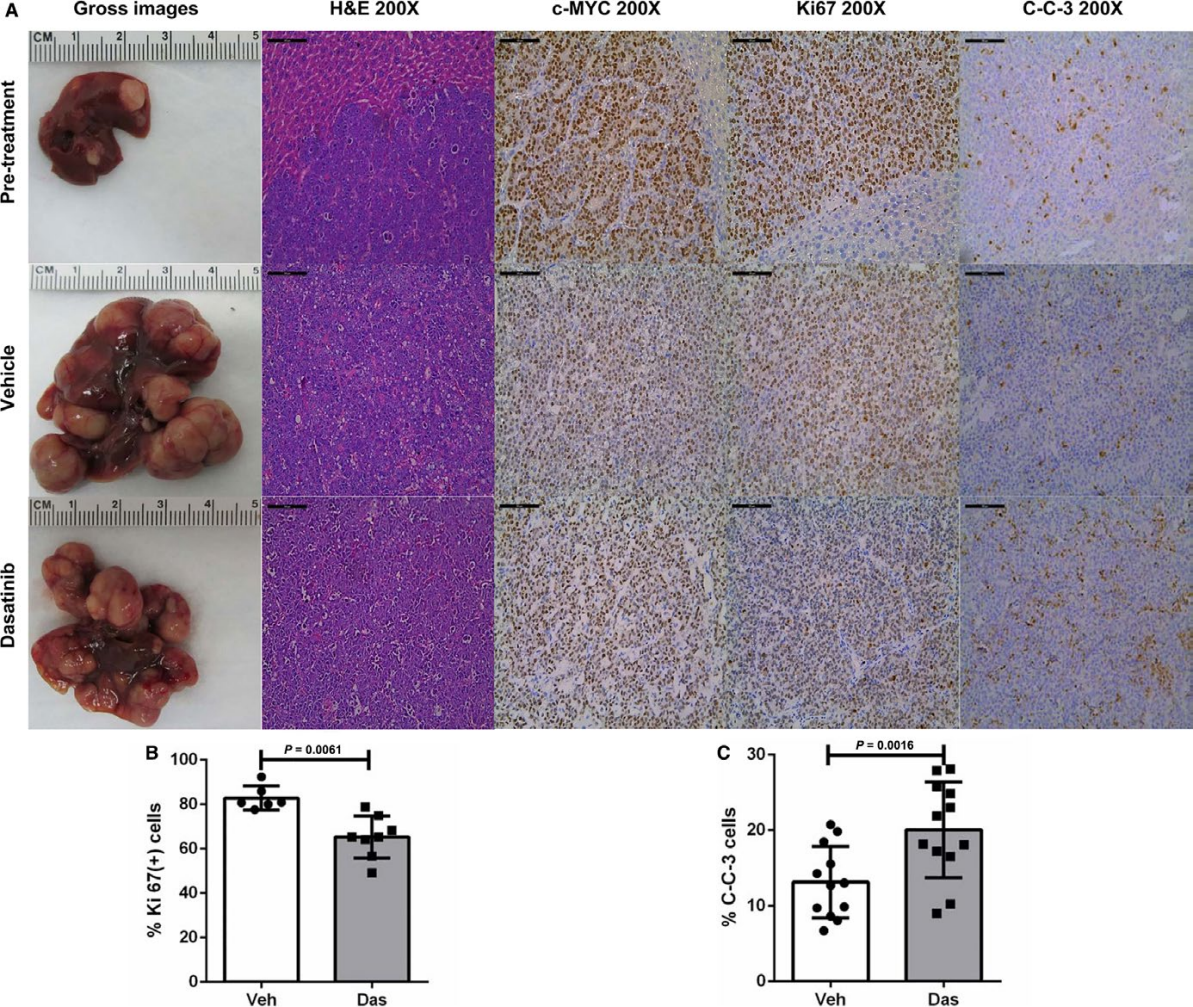
Dasatinib treatment inhibits proliferation and promotes apoptosis in c‐Myc mouse HCC. A, Gross images, H&E staining and immunohistochemical staining of pretreated, vehicle treated, and Dasatinib treated FVB/N mice. Scale bars: 100 μm for H&E, c‐Myc, Ki67 and C‐C‐3 staining. B, Quantification of Ki67 immunostaining. Each dot represents one measurement replicate (Veh, n = 6; Das, n = 8). C, C‐C‐3 apoptosis upon Dasatinib treatment. Each dot represents one measurement replicate (Veh, n = 12; Das, n = 12). Data are presented as mean ± SD; and *P*‐value was calculated using Mann‐Whitney *U* test. C‐C‐3, Cleaved Caspase 3; Das, Dasatinib; SL, surrounding liver; T, tumor; Veh, Vehicle

At the histological level, all tumors consisted of basophilic, poorly differentiated HCC (Figure 4A). All tumor cells (100%) expressed ectopically injected c‐Myc oncoprotein (Figure 4A). Tumor cells were highly proliferative, as assessed by diffuse immunoreactivity for Ki67 staining. Quantification of Ki67 immunostaining revealed that Dasatinib treatment decreased cell proliferation rate compared with vehicle treated mice, although tumor cell proliferation rate remained high (Figure 4B). As concerns cell apoptosis rate, using cleaved caspase 3 as a biomarker, we found that a rise in apoptosis was triggered by Dasatinib treatment (Figure 4A,C).

Altogether, our study demonstrates that Dasatinib is able to induce the decreased cell proliferation and increased apoptosis in c‐Myc mouse HCC. However, the effects were moderate, and tumors continued to grow, although at a slower pace than vehicle treated mice. Therefore, Dasatinib, as a single agent, has limited efficacy against c‐Myc driven HCC.

### 3.4 Dasatinib treatment induces FAK activation in c‐Myc mouse HCC

**Figure 5:**
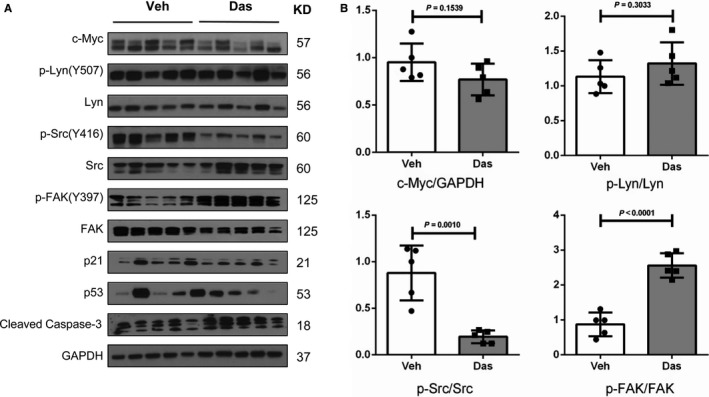
Molecular analysis of Dasatinib treated c‐Myc mouse liver tumor tissues. A, Western blot analysis of c‐Myc, p‐Lyn, p‐Src, and p‐FAK in Vehicle and Dasatinib treated c‐Myc mouse liver tumor tissues. GAPDH was used as loading control. B, Quantification of protein levels of c‐Myc/GAPDH, p‐Lyn/Lyn, p‐Src/Src and p‐FAK/FAK in mouse HCC samples. Data are presented as mean ± SD; and *P*‐value was calculated using Mann‐Whitney *U* test. Each dot represents one biological replicate (Veh, n = 5; Das, n = 5). Das, Dasatinib; Veh, Vehicle

To investigate the mechanisms limiting the efficacy of Dasatinib against c‐Myc driven mouse HCC, we evaluated the expression levels of Dasatinib targets in vehicle or Dasatinib treated mouse HCC samples. We found that Dasatinib treatment effectively inhibited p‐Src levels in the mouse liver, while not affecting p‐Lyn levels (Figure 5A,B). Importantly, we found that, similar to that detected in HCC cell lines, Dasatinib triggered up regulation of p‐FAK in c‐Myc HCC (Figure 5A,B). Other pathways, including Ras/MAPK, AKT/mTOR, p53/p21, and Stat3 and NF‐κB cascades, did not show consistent changes in c‐Myc HCCs upon Dasatinib treatment (Figure S4).

In summary, the present data indicate that Dasatinib treatment induces upregulation of p‐FAK in c‐Myc HCC, which may contribute to the limited efficacy of Dasatinib in HCC treatment.

### 3.5 Combined treatment of Dasatinib with FAK inhibitor resulted in strong growth inhibition in HCC cell lines

**Figure 6:**
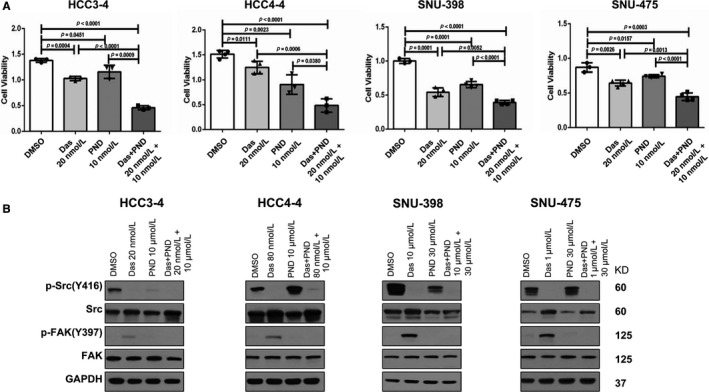
Combined treatment of Dasatinib and PND‐1186 results in strong growth inhibition in HCC cell lines. A, Cell viability of HCC3‐4, HCC4‐4, SNU‐398, SNU‐475 cells when treated with Dasatinib, PND‐1186, or Dasatinib+PND‐1186 at ~IC50 concentration determined using crystal violet staining. Data are presented as mean ± SD; and *P*‐value was calculated using Mann‐Whitney *U* test. Each dot represents one treatment replicate. B, Expression of p‐Src, Src, p‐FAK and FAK in HCC cell lines analyzed using Western blotting. GAPDH was used as loading control. Das, Dasatinib; Veh, Vehicle

**Figure 7:**
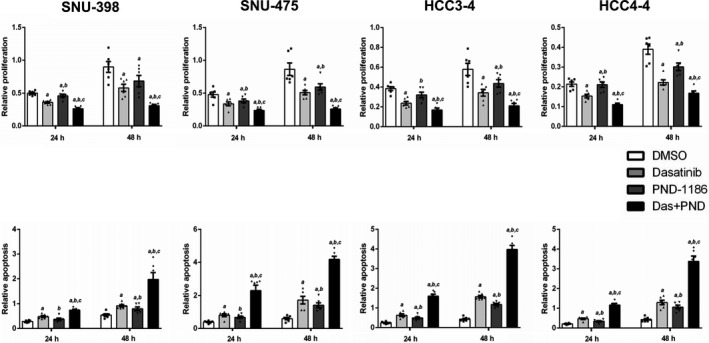
Relative proliferation and apoptosis after Dasatinib, PND‐1186 or Dasatinib and PND‐1186 treatment at different time courses (24 and 48 h) in four HCC cell lines (SNU‐398, SNU‐475, HCC3‐4 and HCC4‐4). Cell proliferation rate was determined using the BrdU Cell Proliferation Assay; whereas cell apoptosis rate was analyzed using the Cell Death Detection Elisa Assay. Upper panels: proliferation; lower panels: apoptosis. Data are presented as mean ± SD. Mann‐Whitney *U* test, *P* < 0.05 (a) vs DMSO; (b) vs Dasatinib; (c) vs PND‐1186. See Table S4 for the specific *P* values for each comparison. Each dot represents one treatment replicate

Our in vitro and in vivo studies suggest that up‐regulation of FAK activity might contribute to the poor responsiveness to Dasatinib of HCC cells. Therefore, we tested the hypothesis that FAK inhibitor may prevent Dasatinib induced up regulation of p‐FAK and together with Dasatinib lead to increased HCC cell growth inhibition. For this purpose, we chose PND‐1186, also known as VS‐4718. PND‐1186 is a potent FAK inhibitor with good oral bioavailability^28^ and it has been shown to inhibit tumor growth and metastasis in pre‐clinical models.^29^ We tested IC_50_ against PND‐1186 in HCC3‐4 and HCC4‐4 mouse c‐Myc HCC cell lines as well as two randomly selected human HCC cell lines, SNU475 and SNU398 cells using cell viability assays. We found that PND‐1186 effectively inhibited HCC cell growth with IC_50_ ~ 5 to 25 μmol/L (Figure S5). We next treated HCC3‐4, HCC4‐4, SNU475 and SNU398 HCC cells with Dasatinib, PND‐1186, or Dasatinib+PND‐1186. While Dasatinib or PND‐1186 administration alone was able to inhibit HCC cell growth, combined Dasatinib and PND‐1186 treatment resulted in the highest growth inhibition in all 4 cell lines tested (Figure 6A). At the molecular levels, while Dasatinib treatment induced FAK activation, this increase of p‐FAK expression was efficiently inhibited by PND‐1186 when the two drugs were simultaneously added to the culture medium (Figure 6B). Dasatinib and PND‐1186 combination did not lead to consistent changes of AKT/mTOR, MAPK or Stat3 pathways (Figure S6). Intriguingly, combined Dasatinib and PND‐1186 treatment resulted in significant decrease of c‐Myc expression levels in all 4 cell lines tested (Figure S6). At the cellular level, the synergistic activity of Dasatinib and PND‐1186 resulted in strong decrease of proliferation and induction of apoptosis in the four cell lines (Figure 7).

In summary, our current results suggest that FAK inhibitors suppress Dasatinib induced FAK activation, and combined treatment with Dasatinib and FAK inhibitors may represent a novel therapeutic strategy for HCC.

## 4 DISCUSSION

Overexpression and/or amplification of c‐Myc oncogene is one of the most frequent genetic alterations in multiple tumor types, including human HCC. A wealth of evidence has demonstrated that c‐Myc is a difficult druggable target. In recent years, much effort has been devoted to applying synthetic lethal approach to identify molecules that can be targeted to kill c‐Myc tumor cells. For instance, it has been shown that c‐Myc tumors are highly dependent on glutamine, and targeting glutaminase (GLS) may be particularly effective against c‐Myc induced tumors.^30^ However, most of these molecules leading to c‐Myc dependent synthetic lethality are still undergoing early stage clinical evaluation. Also, they are not FDA approved drugs, and their application as effective cancer drugs in humans remains unknown. In a recent study, Martins et al^16^ described Dasatinib as a potent inhibitor of c‐Myc activated cancer cells. Mechanistically, it was found that Dasatinib synthetic lethality was mediated by inhibition of p‐Lyn in c‐Myc overexpressing tumor cells. While Dasatinib has been studied in HCC cell lines,^12^, ^18^ whether Dasatinib is effective against c‐Myc over expressing HCC cells and whether c‐Myc and/or p‐Lyn could be used as biomarker for Dasatinib sensitivity in HCC have not been previously characterized.

In the first part of the study, we discovered that the sensitivity to Dasatinib is highly assorted in HCC cell lines. In addition, while Dasatinib treatment was able to effectively inhibit p‐Lyn and p‐Src expression in HCC cells, the antineoplastic activity of Dasatinib was not correlated to c‐Myc, p‐Lyn, or p‐Src levels. Thus, our in vitro data do not support the hypothesis that the baseline levels of c‐Myc, p‐Lyn, or p‐Src could be used as biomarker to predict Dasatinib responsiveness, at least in HCC cells.

Using hydrodynamic transfection, we previously established a murine HCC model for c‐Myc induced HCC.^13^, ^27^ This preclinical model has been useful to dissecting the genetic alterations, signaling pathways, and metabolic cascades required for Myc‐induced hepatocarcinogenesis, as well as for testing drugs that may be useful for treating c‐Myc positive HCC.^27^, ^31^, ^32^, ^33^, ^34^, ^35^ In the present investigation, we tested the therapeutic potential of Dasatinib for c‐Myc induced HCC using this preclinical model. We found that Dasatinib treatment induced decreased cell proliferation and increased apoptosis, leading to reduced liver tumor burden when compared to vehicle treated mice. However, the decreased proliferation and increased apoptosis were clearly not sufficient to overcome the high proliferation properties of c‐Myc tumor cells. The net outcome was that c‐Myc tumors continued to grow despite Dasatinib therapy, leading to increase tumor burden in Dasatinib treated cohort than that in pretreatment cohort. Overall, our study demonstrates that Dasatinib has limited therapeutic efficacy as a single agent, and it is unable to constrain the aggressive and fast growth of c‐Myc HCC in vivo.

We noticed that despite that Dasatinib was able to consistently inhibit p‐Lyn in human HCC cell lines, Dasatinib at the dose we were using, did not lead to significant decreased p‐Lyn expression in c‐Myc mouse HCC samples. The results highlight the fact that tumor cells may depend on distinct signaling cascades and may respond to drugs in a drastically different manner in vitro and in vivo. The difference may be due to the fact that cells cultured in vitro have higher and consistent exposure to the drug; whereas cells in vivo may have rather limited exposure and the drug is continuously eliminated from the body. These mechanisms may contribute to fact that many drugs demonstrate excellent tumor growth inhibitory activities in cell culture system, yet they have limited, if any therapeutic efficacy in humans. It is worth to mention that Dasatinib has been tested in a phase II clinical trial for patients with advanced HCC (NCT00459108). The study was terminated early for the lack of efficacy. While patient sample size was very small for the clinical trial (n = 25, with only 14 patients able to complete the study), the collected data suggest that, in unselected HCC patients, Dasatinib is unlikely to be useful. Our current preclinical study is consistent with the clinical observation. The results also support the use of preclinical models to study drug therapy for cancer treatment.

Despite the inability of Dasatinib to inhibit HCC development in c‐Myc mice, it is important to note that it was still able to decrease HCC cell proliferation and increase apoptosis. This finding suggests the possibility that Dasatinib could be efficacious when used in combination with other drugs to enhance the inhibitory activity on tumor growth. Importantly, we found that Dasatinib treatment consistently triggered FAK activation in all HCC cell lines tested as well as in the c‐Myc mouse HCC model. FAK has been found to be over expressed in human HCC samples. It has a major role in regulating HCC cell growth and metastasis,^36^ and is involved in resistance to drugs, such as Sorafenib.^37^ It is worth to note that both c‐Myc and FAK are major oncogenic signals during tumorigenesis. Previous studies have implicated c‐Myc and FAK in cancer initiation, progression and resistant to chemotherapy. For example, it was found that inhibition of FAK and c‐Myc synergized to suppress ovarian cancer cell growth^38^; and c‐Myc and FAK functioned together to promote invasion of skin cancer cells.^39^ Our study uncovered a novel biochemical crosstalk between c‐Myc and FAK activation in HCC cells in response to Dasatinib. The results suggest that the activation of FAK may limit the therapeutic efficacy of Dasatinib in c‐Myc driven HCC. Thus, it is possible that the activation of FAK may confer resistance to Dasatinib. We tested this hypothesis by using a FAK inhibitor, PND‐1186. We noted that PND‐1186 appeared to have relative moderate activity in suppressing HCC cell proliferation when compared with Dasatinib as a single agent (Figure 7A). The moderate growth suppressive effects of FAK inhibitor were consistent with the results obtained in human HCC cells subjected to FAK silencing using specific shRNA.^40^ Importantly, PND‐1186 effectively inhibited Dasatinib induced p‐FAK expression, and it synergized with Dasatinib to inhibit HCC cell growth in vitro by decreasing proliferation and inducing apoptosis. The results suggest that combined Dasatinib and FAK inhibitors might represent novel therapeutic agents against HCC.

Clearly, additional studies are required to characterize the molecular mechanisms by which Dasatinib activates FAK in HCC cells. It is well‐known that FAK could be activated by integrin family members. It is therefore important to analyze whether Dasatinib treatment induces the overexpression and/or activation of integrin(s) in HCC cells. Furthermore, since Dasatinib consistently inhibits SRC activities in vitro and in vivo, activation of FAK could be a feedback mechanism in response to the loss of SRC activity. This hypothesis should be further substantiated by deleting SRC kinase in HCC cell lines using CRISPR/Cas9 based gene editing technology. Our in vitro experiments support the combined administration of Dasatinib and FAK inhibitors for HCC treatment. Obviously, the therapeutic effectiveness as well as the potential toxicity of the combined therapy need to be tested in vivo, preferably using both genetic engineered mouse HCC model as well as patient‐derived HCC xenografts. These studies would be highly helpful to determine whether the combination therapy of Dasatinib and FAK inhibitors should be evaluated in clinical trials for HCC patients.

## CONFLICTS OF INTEREST

The authors declare no conflict of interest.
