# Supplementary material for: Focal adhesion kinase activation limits efficacy of Dasatinib in c‐Myc driven hepatocellular carcinoma
Source: Cancer Med. 2018 Oct 28;7(12):6170–81. doi: 10.1002/cam4.1777 (PMC6308083; doi:10.1002/cam4.1777)
Supplement: Supplementary file 1 [file CAM4-7-6170-s001.pdf]

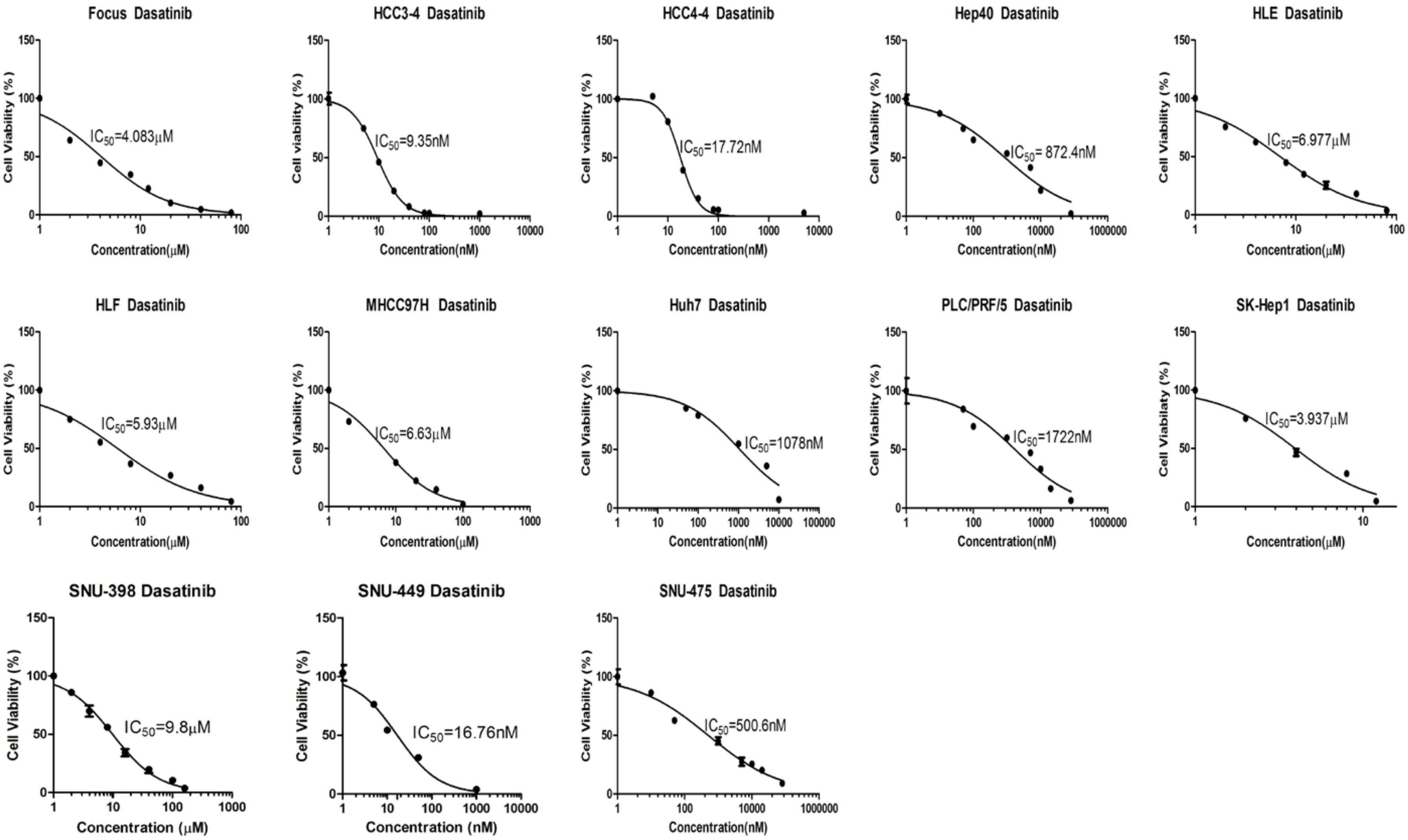

Supporting Information Figure S1. IC50 against Dasatinib in a panel of HCC cell lines (Focus, Hep40, HLE, HLF MHCC97H, Huh7, PLC/PRF/5, SK-HEP1, SNU-398, SNU-449, SNU-475) and 2 mouse HCC cell lines (HCC3-4 and HCC4-4).

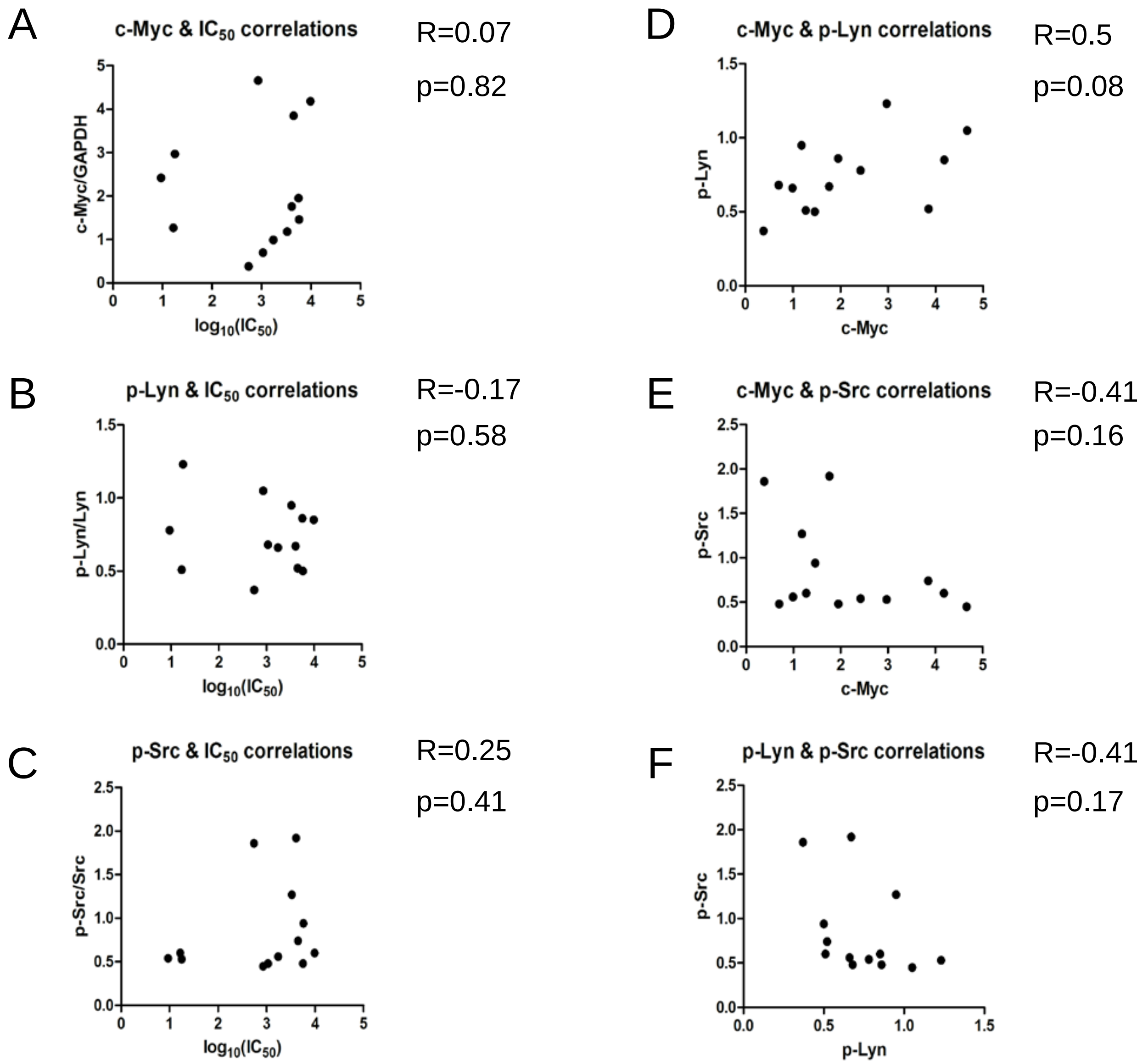

Supporting Information Figure S2. Correlation between Dasatinib IC50 values and c-Myc, p-Lyn, and p-Src expression levels in HCC cell lines. Correlation between: (A) c-Myc expression and Dasatinib IC50; (B) p-Lyn expression and Dasatinib IC50; (C) p-Src expression and Dasatinib IC50; (D) c-Myc and p-Lyn expression in HCC cells; (E) c-Myc and p-Src expression; (F) p-Lyn and p-Src expression.

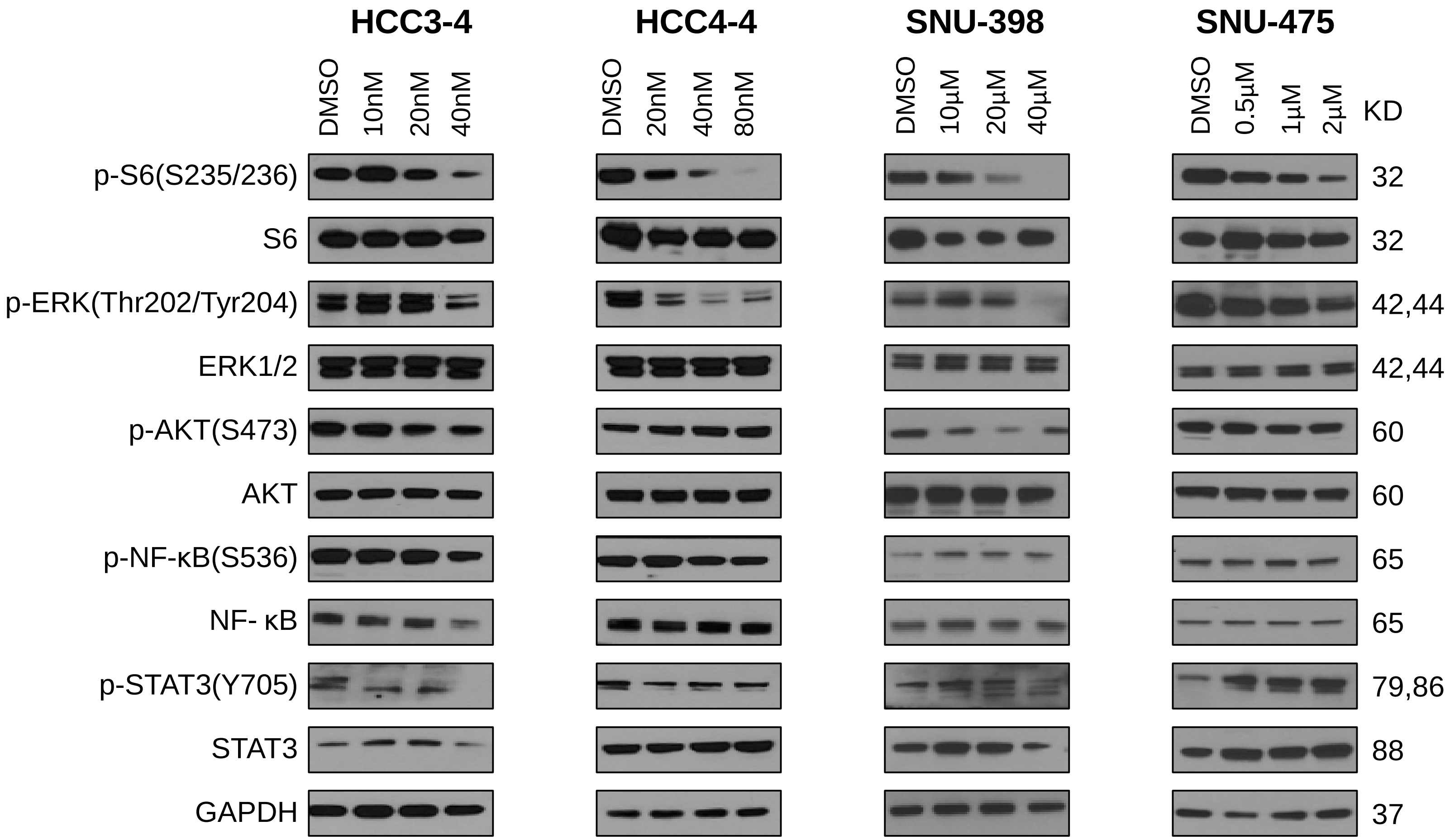

Supporting Information Figure S3. Molecular analysis of Dasatinib treated HCC cell lines. HCC3-4,HCC4-4, SNU-398 and SNU-475 cells were treated with Dasatinib at IC50 concentration, and expression of p-S6, S6, p-ERK, ERK, p-AKT, AKT, p-NF-κB, NF-κB, p-STAT3 and STAT3 was analyzed using Western blotting. GAPDH was used as loading control.

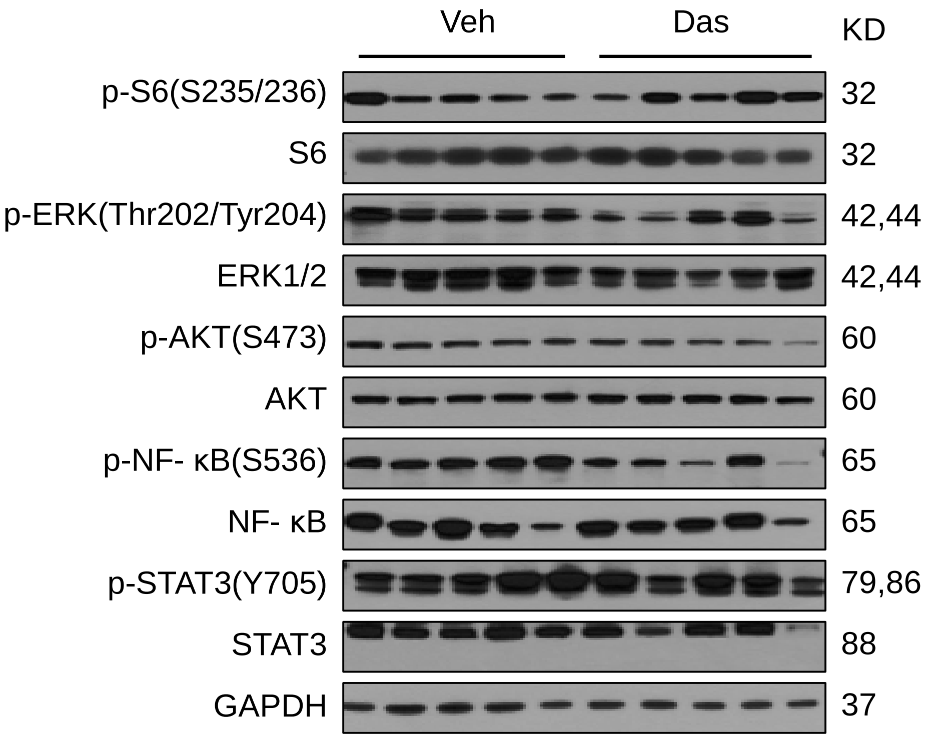

Supporting Information Figure S4. Molecular analysis of Dasatinib treated c-Myc mouse liver tumor tissues. Western blot analysis of of p-S6, S6, p-ERK, ERK, p-AKT, AKT, p-NF-κB, NF-κB, p-STAT3 and STAT3 in Vehicle and Dasatinib treated c-Myc mouse liver tumor tissues. GAPDH was used as loading control. Abbreviations: Das, Dasatinib; Veh, vehicle.

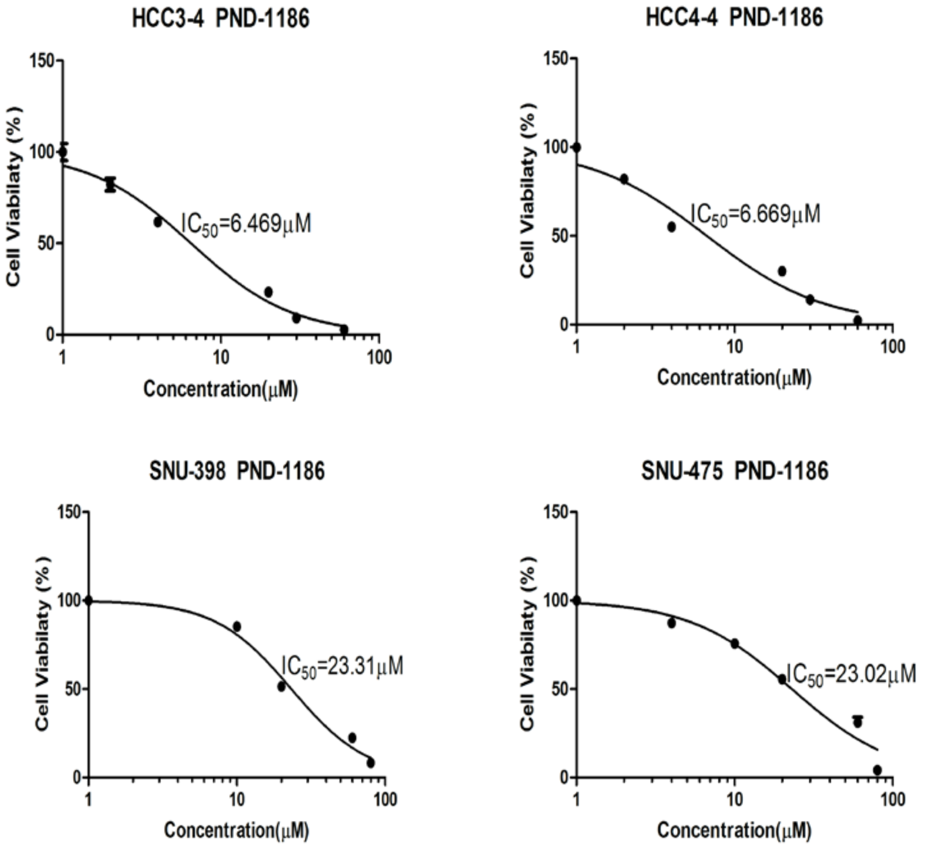

Supporting Information Figure S5. IC50 against PND-1186 in a panel of HCC cell lines (HCC3-4, HCC4-4, SNU-398 and SNU-475).

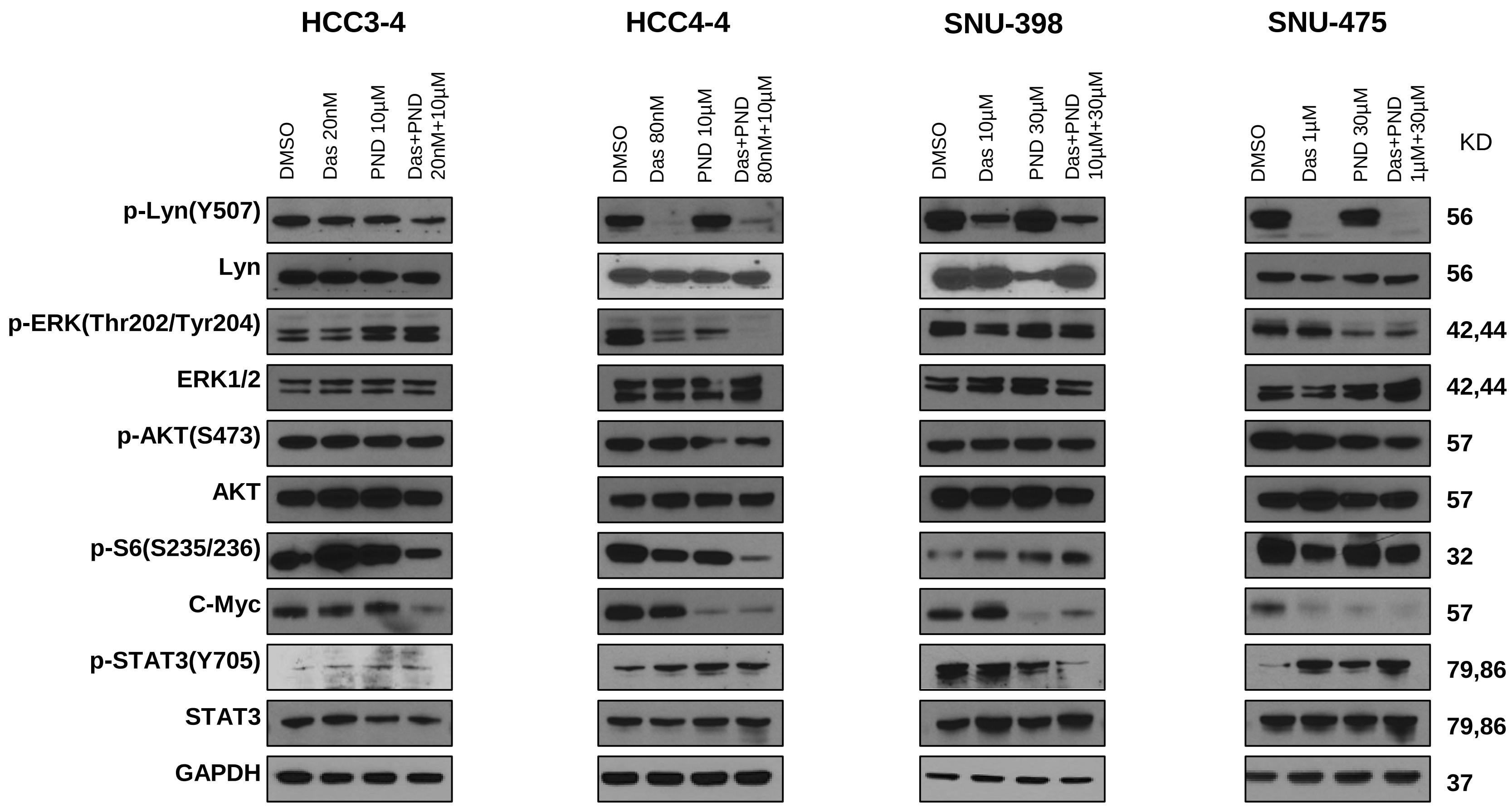

Supporting Information Figure S6. Molecular analysis of Dasatinib and/or PND-1186 treated HCC cell lines.

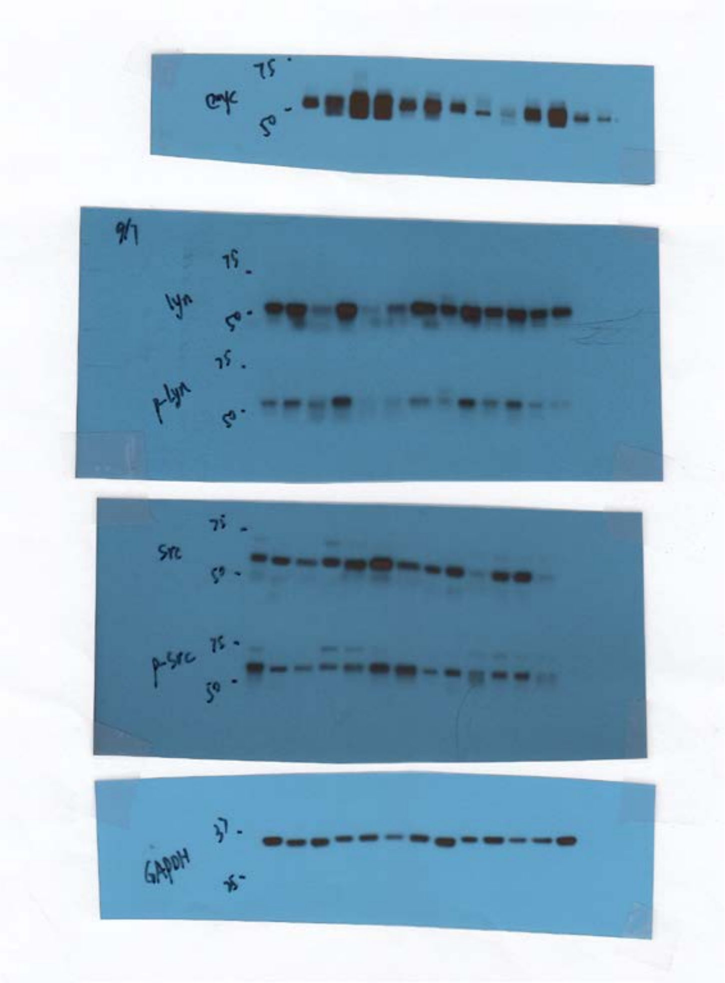

Supporting Information Figure S7. Original gel images for the Western blots of Figure 1.

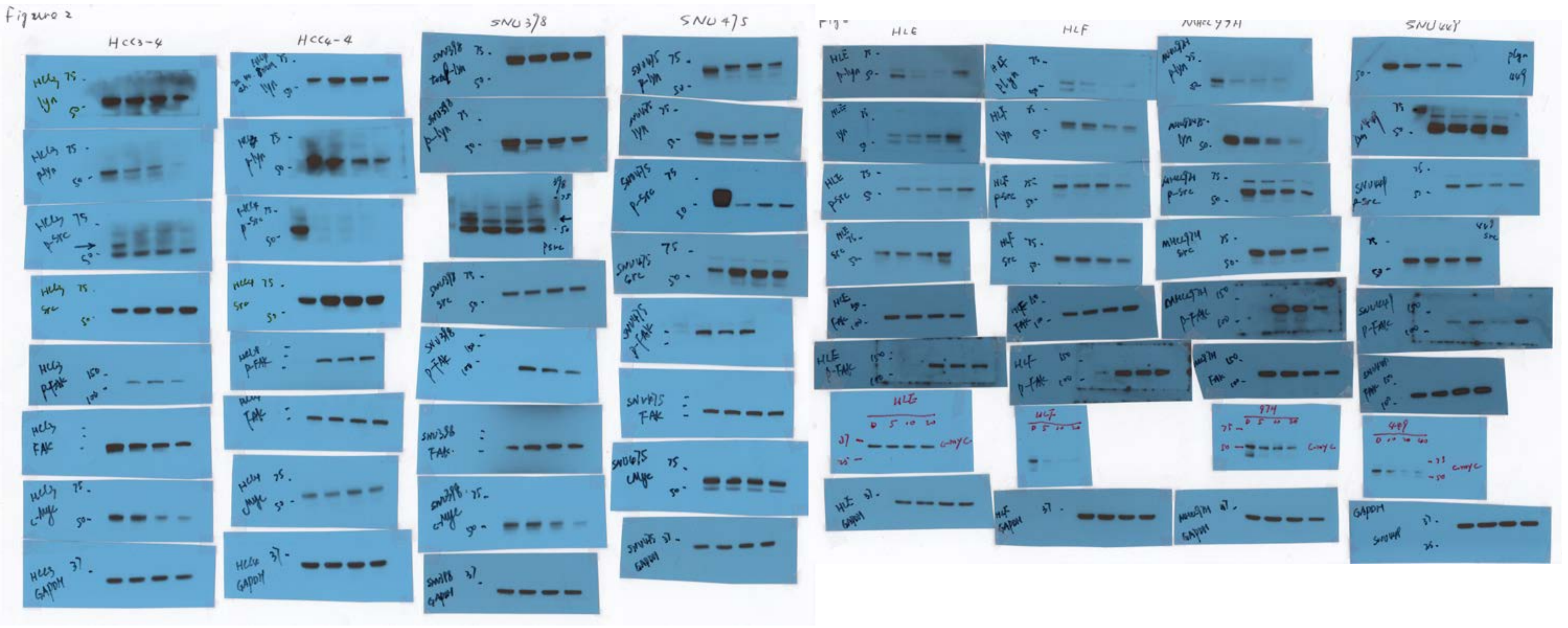

Supporting Information Figure S8. Original gel images for the Western blots of Figure 2.

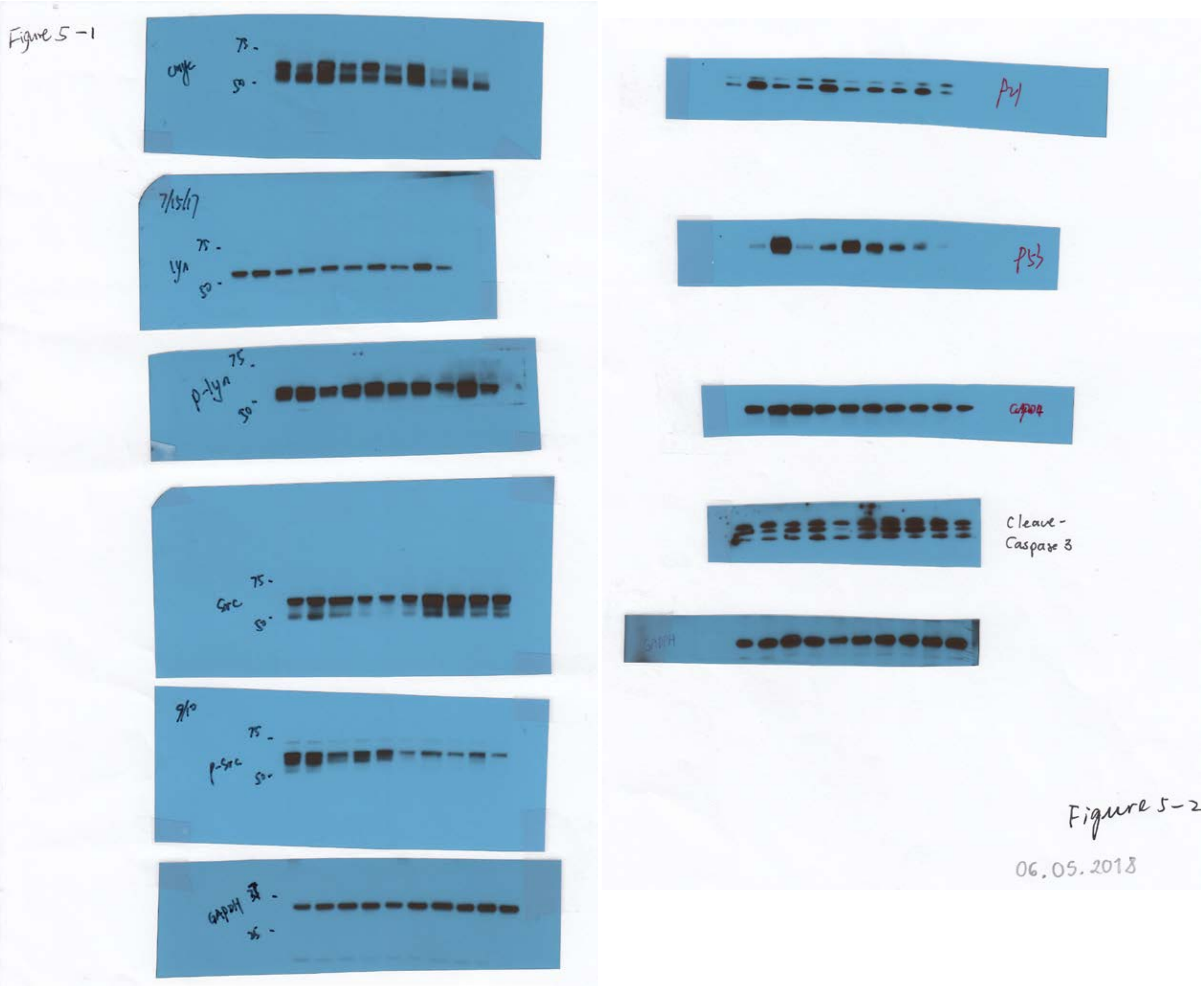

Supporting Information Figure S9. Original gel images for the Western blots of Figure 5.

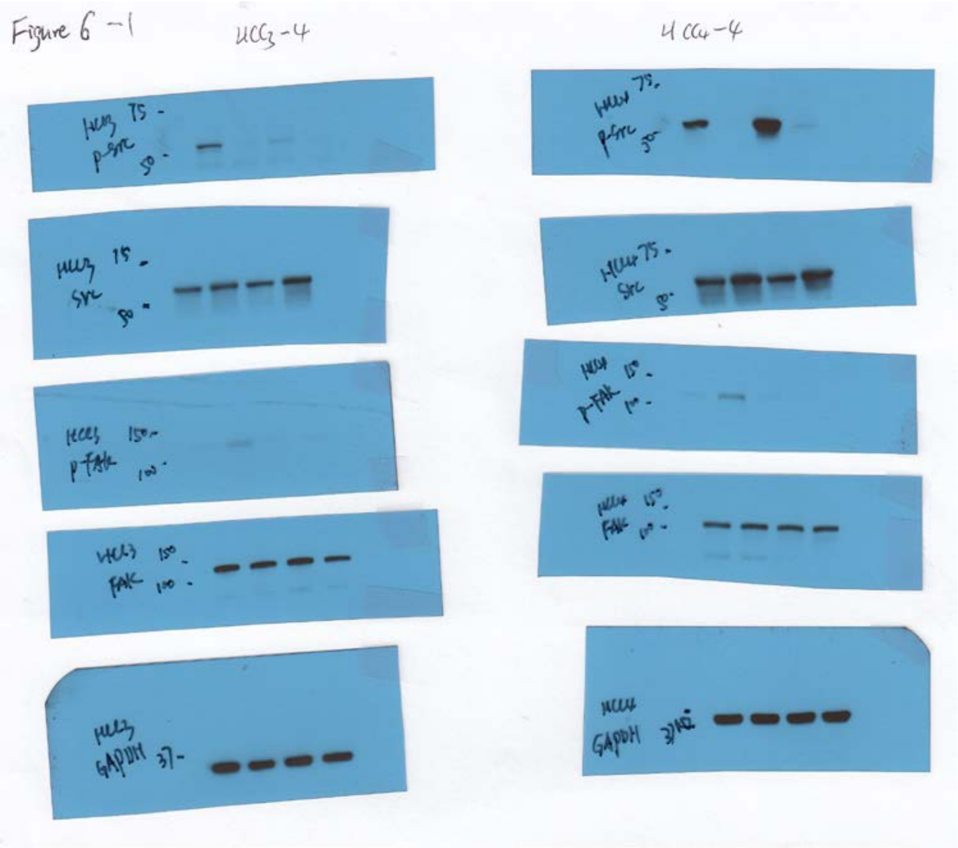

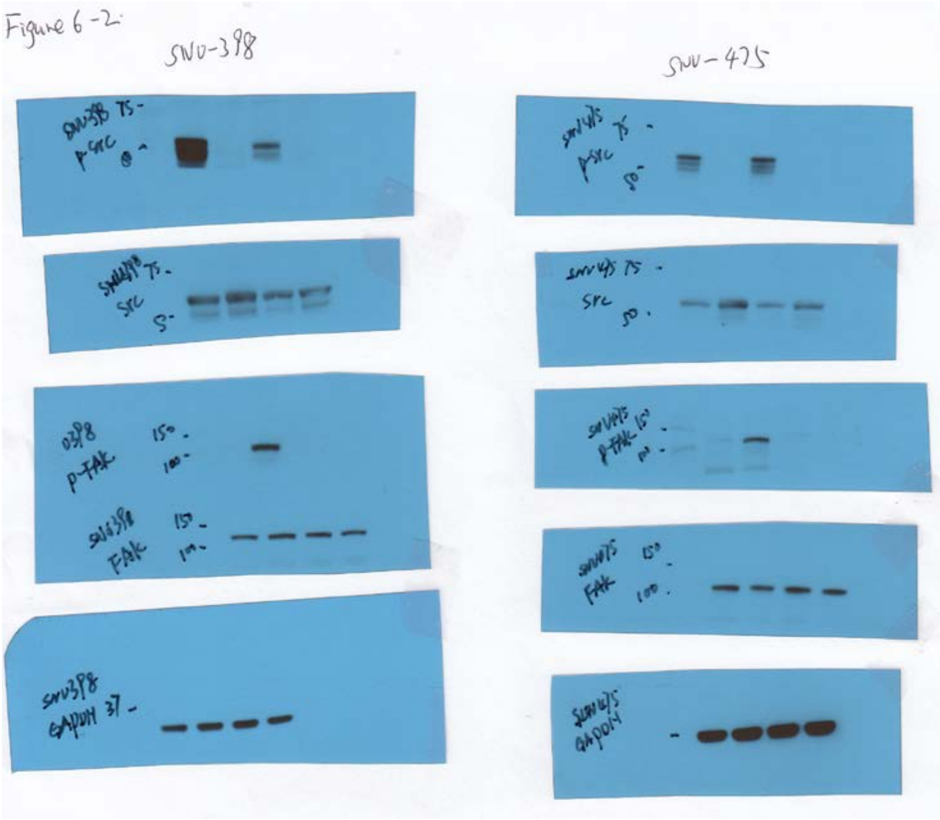

Supporting Information Figure S10. Original gel images for the Western blots of Figure 6.

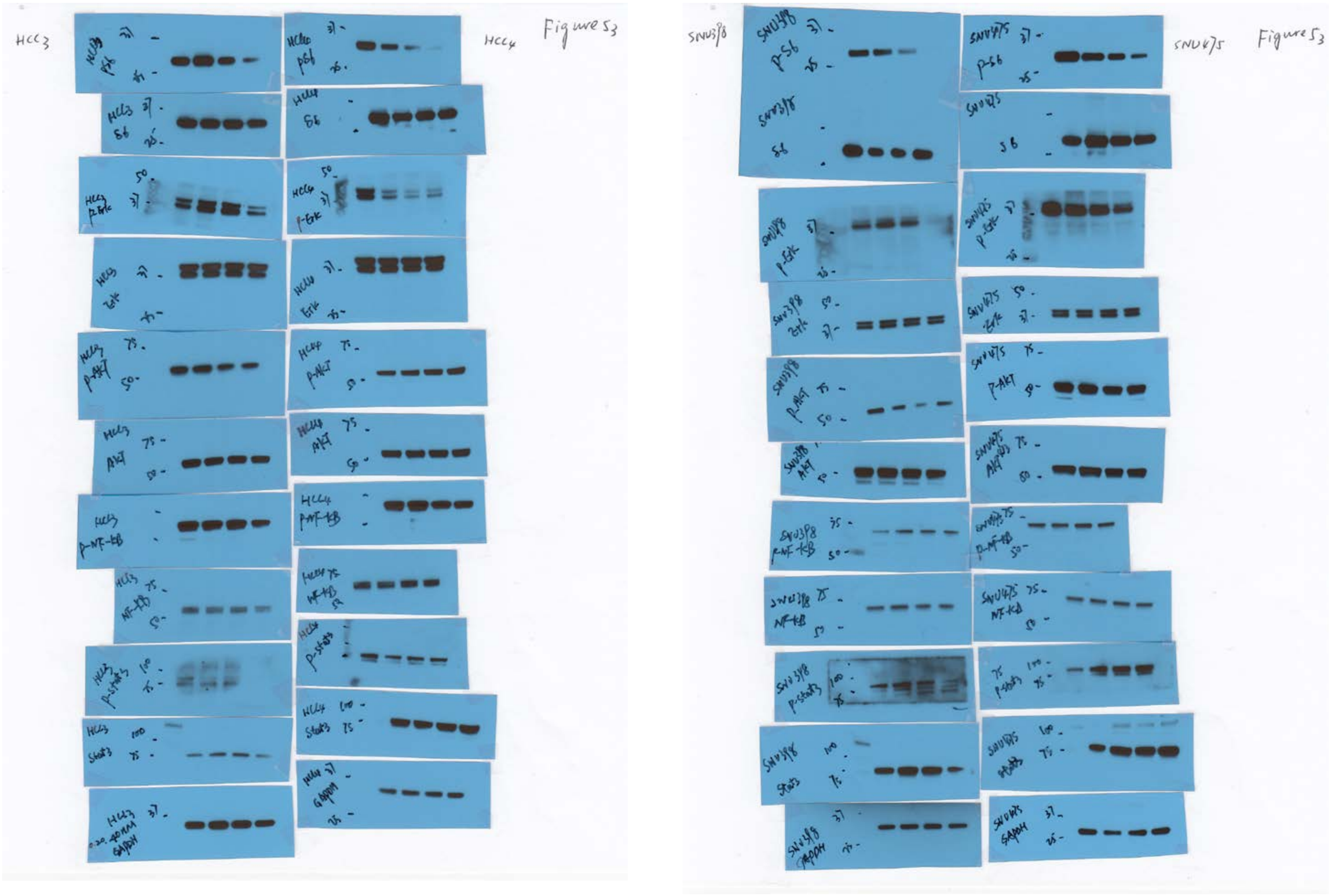

Supporting Information Figure S11. Original gel images for the Western blots of Figure S3.

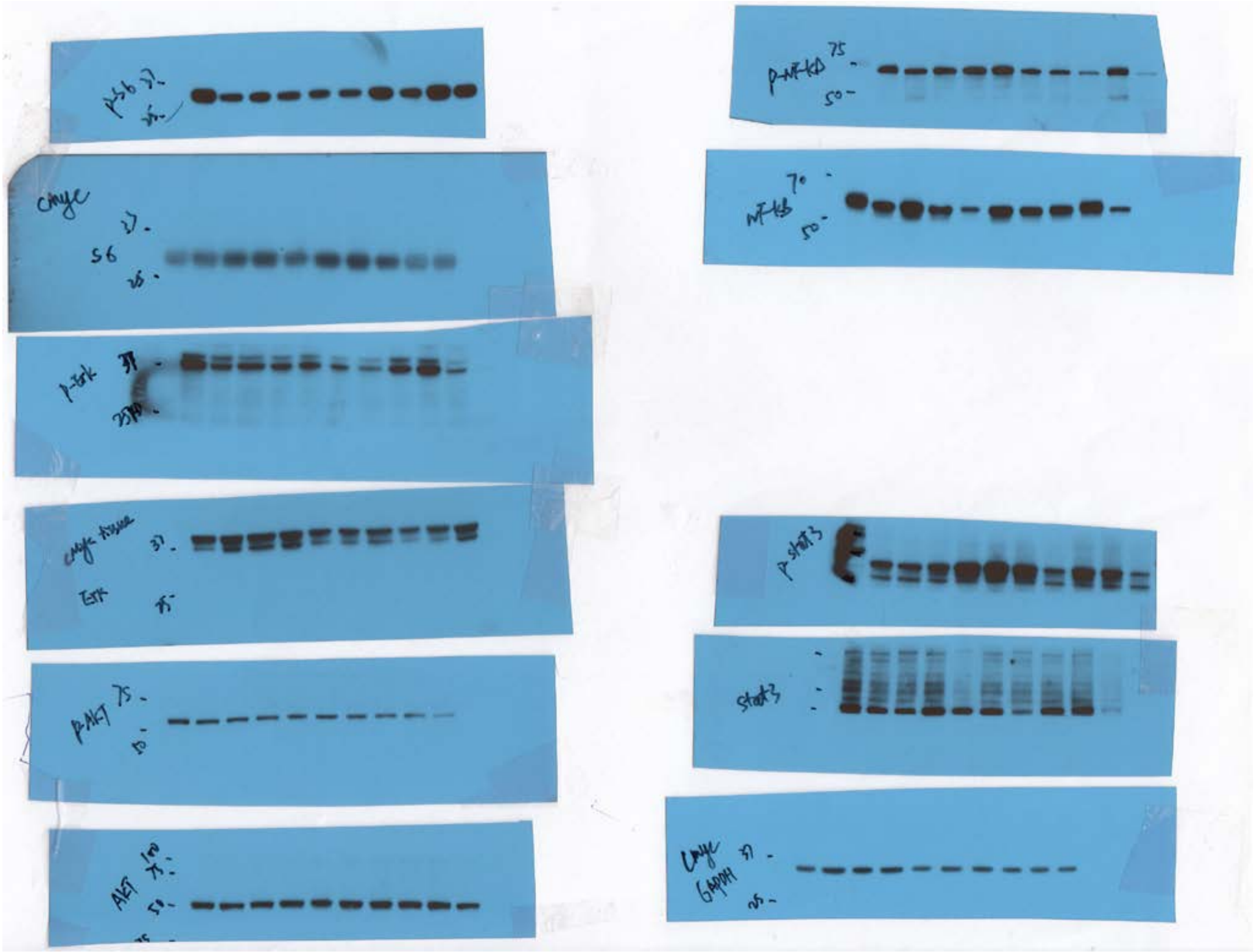

Supporting Information Figure S12. Original gel images for the Western blots of Figure S4.

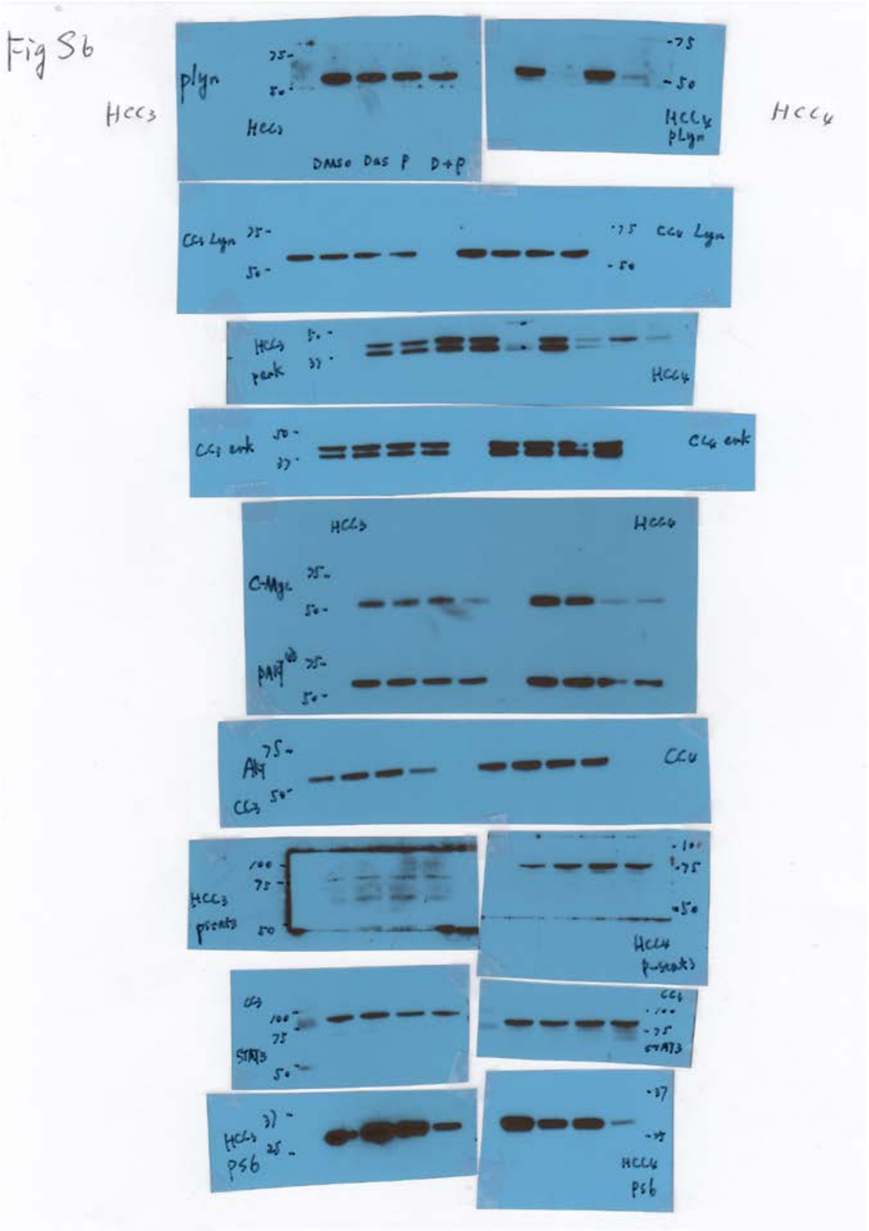

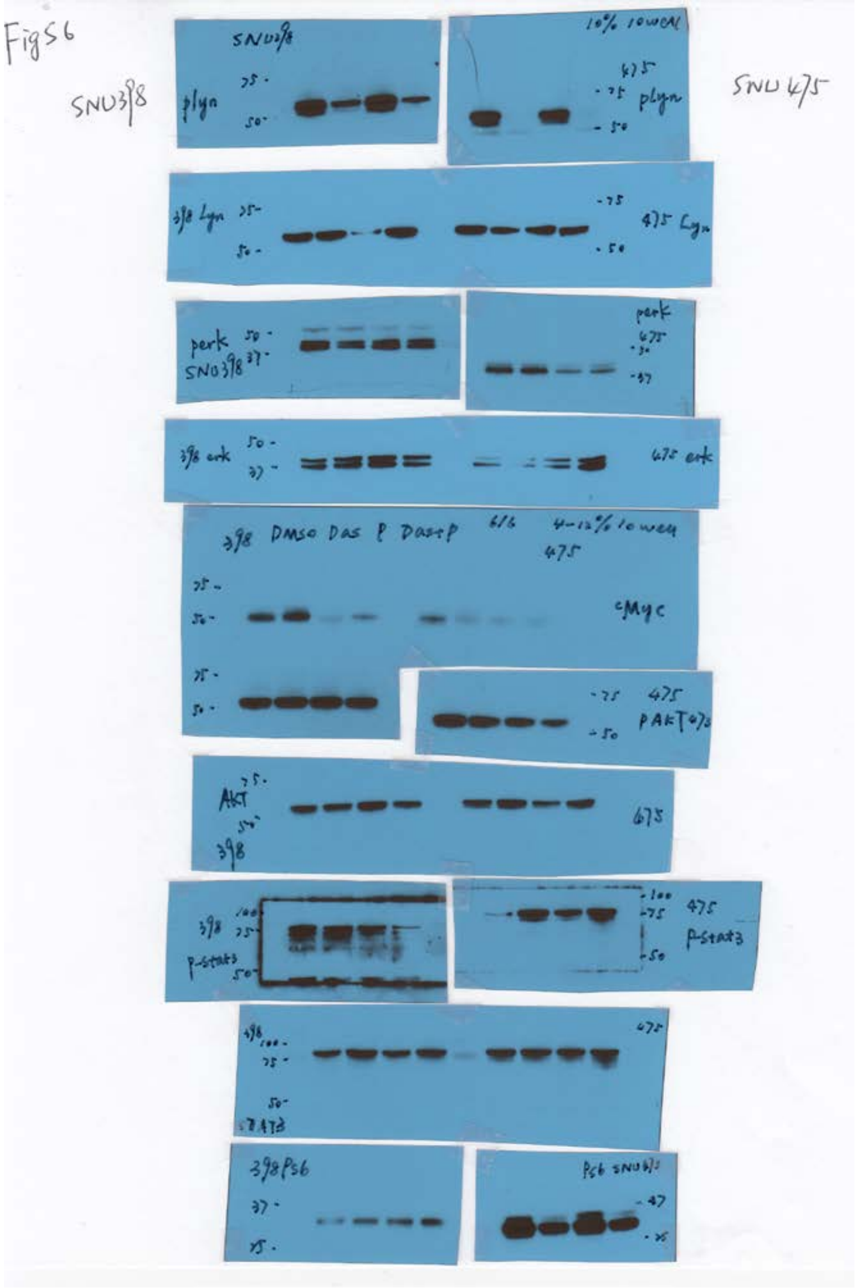

Supporting Information Figure S13. Original gel images for the Western blots of Figure S6.
