# Supplementary material for: Focal adhesion kinase activation limits efficacy of Dasatinib in c‐Myc driven hepatocellular carcinoma
Source: Cancer Med. 2018 Oct 28;7(12):6170–81. doi: 10.1002/cam4.1777 (PMC6308083; doi:10.1002/cam4.1777)
Supplement: Supplementary file 2 [file CAM4-7-6170-s002.docx]

|  |  |  |
| --- | --- | --- |
| **Reagents and Kits** | **Company** | **Catalog number** |
| The Endotoxin Free Maxi Prep Kit | Sigma-Aldrich | NA0410-1 KT |
| Dasatinib | LC Laboratories | CAY22368 |
| PND-1186 | Selleckchem | S7653 |
| DMSO | ThermoFisher Scientific | BP231 |
| Zinc formal-fix | ThermoFisher Scientific | 6764255 |
| Hematoxylin | ThermoFisher Scientific | SH26-500D |
| Eosin | ThermoFisher Scientific | SE23-500D |
| Avidin-Biotin Blocking Kit | Vector Laboratories | SP-2001 |
| Vectastain ABC Elite Kit | Vector Laboratories | SK-4105 |
| Mammalian Protein Extraction Reagent | ThermoFisher Scientific | 78501 |
| Complete Protease Inhibitor Cocktail | Roche Molecular Biochemicals | 1861281 |
| Protein Assay Kit | Bio-Rad | 23225, 23227 |
| SDS-PAGE gel | Bio-Rad | 456-1043 |
| nitrocellulose membranes | Bio-Rad | 162-0167 |
| horseradish peroxidase-secondary antibody | Jackson Immuno Research Laboratories Inc. | 115-035-003(anti-mouse) ; 111-035-144(anti-rabbit) |
| Super Signal West Femto | Pierce Chemical Co. | 34075; 34094 |
| fetal bovine serum | Gibco | 12306C-100ML |
| Penicillin and streptomycin | Sigma-Aldrich | P4333-100ML |
| crystal violet | Sigma-Aldrich | C6158-50G |
| BrdU Cell Proliferation Assay Kit | Cell Signaling Technology | BDB559619 |
| Cell Death Detection Elisa Plus Kit | Roche Molecular Biochemicals | 11544675001 |
|  |  |  |

**Supporting Information Table S1: Reagents and Kits Information**

**Supporting Information Table S2: Immunocytochemistry and Western blotting Antibody Information**

|  |  |  |  |  |
| --- | --- | --- | --- | --- |
| Antibody | Company | Catalog number | Dilution | Method |
| c-Myc(Y69) | Abcam | ab32072 | 1:200 | IHC |
| Ki67(SP6) | Thermo Scientific | MA5-14520 | 1:100 | IHC |
| Cleaved-caspase-3(D175) | Cell signaling technology | 9664 | 1:300 | IHC |
| c-Myc(Y69) | Abcam | ab32072 | 1:5000 | WB |
| Phospho-Lyn(Tyr507) | Cell signaling technology | 2731 | 1:1000 | WB |
| Lyn(C13F9) | Cell signaling technology | 2796 | 1:1000 | WB |
| Phospho-Src Family(Tyr416) | Cell signaling technology | 6943T | 1:1000 | WB |
| Src(36D10) | Cell signaling technology | 2109T | 1:1000 | WB |
| GAPDH(D16H11) | Cell signaling technology | 5174 | 1:5000 | WB |
| YAP/TAZ(D24E4) | Cell Signaling Technology | 8418 | 1:1000 | WB |
| Phospho-NF-kBp65(S536) | Cell Signaling Technology | 3033 | 1:1000 | WB |
| NF-kBp65 | Cell Signaling Technology | 8242 | 1:1000 | WB |
| Phospho-FAK(Y397) | Cell Signaling Technology | 3283 | 1;1000 | WB |
| FAK | Cell Signaling Technology | 3285 | 1:1000 | WB |
| Phospho-STAT3(Y705) | Cell Signaling Technology | 9145 | 1:1000 | WB |
| STAT3 | Abcam | ab68153 | 1:1000 | WB |
| Phospho-ERK(p44/42) | Cell Signaling Technology | 4370 | 1:1000 | WB |
| ERK1/2 | Cell Signaling Technology | 9102 | 1:1000 | WB |
| Phospho-AKT(S473) | Cell Signaling Technology | 3787 | 1:1000 | WB |
| AKT | Cell Signaling Technology | 9272 | 1:1000 | WB |
| Phospho-RPS6(S235/236) | Cell Signaling Technology | 4858 | 1:2000 | WB |
| S6 | Cell Signaling Technology | 2217 | 1:1000 | WB |
|  |  |  |  |  |

**Supporting Information Table S3: Cell Lines Information**

| **Cell Lines** | **Cell Lines Type** | **Order Information** | **Catalog Number** | **Culture Condition** |
| --- | --- | --- | --- | --- |
| Focus | Human HCC | Dr. Ju-Seog Lee (1) | N/A | DMEM+10%FBS+Pen/Strep |
| HCC3-4 | Mouse c-Myc HCC | Dr. Dean Felsher (2) | N/A | DMEM+10%FBS+Pen/Strep |
| HCC4-4 | Mouse c-Myc HCC | Dr. Dean Felsher (2) | N/A | DMEM+10%FBS+Pen/Strep |
| Hep40 | Human HCC | Dr. Brian Carr (3) | N/A | DMEM+10%FBS+Pen/Strep |
| HLE | Human HCC | JCRB Cell Bank | JCRB0405 | DMEM+10%FBS+Pen/Strep |
| HLF | Human HCC | JCRB Cell Bank | JCRB0404 | DMEM+10%FBS+Pen/Strep |
| MHCC97H | Human HCC | Dr. Binbin Liu (4) | N/A | DMEM+10%FBS+Pen/Strep |
| Huh7 | Human HCC | JCRB Cell Bank | JCRB0403 | DMEM+10%FBS+Pen/Strep |
| PLC/PRF/5 | Human HCC | ATCC | CRL-8024 | DMEM+10%FBS+Pen/Strep |
| SK-HEP1 | Human HCC | ATCC | HTB-52 | DMEM+10%FBS+Pen/Strep |
| SNU398 | Human HCC | ATCC | CRL-2233 | DMEM+10%FBS+Pen/Strep |
| SNU449 | Human HCC | ATCC | CRL-2234 | DMEM+10%FBS+Pen/Strep |
| SNU475 | Human HCC | ATCC | CRL-2236 | DMEM+10%FBS+Pen/Strep |

(1) PubMed ID: 6086498

(2) PubMed ID: 21262914

(3) PubMed ID: 7590653

(4) PubMed ID: 10555751

**Supporting Information Table S4: *p*-values for multi-group comparison in Figure 7**

|  | **SNU398** | | | | **SNU475** | | | | **HCC3-4** | | | | **HCC4-4** | | | | |
| --- | --- | --- | --- | --- | --- | --- | --- | --- | --- | --- | --- | --- | --- | --- | --- | --- | --- |
|  | **24h** | | **48h** | | **24h** | | **48h** | | **24h** | | **48h** | | **24h** | | **48h** | | |
|  | proliferation | apoptosis | proliferation | apoptosis | proliferation | apoptosis | proliferation | apoptosis | proliferation | apoptosis | proliferation | apoptosis | proliferation | apoptosis | proliferation | apoptosis |  |
| **DMSO vs Das (a)** | **<0.0001** | **0.0006** | **<0.0001** | **<0.0001** | **<0.0001** | **<0.0001** | **<0.0001** | **<0.0001** | **<0.0001** | **<0.0001** | **<0.0001** | **<0.0001** | **<0.0001** | **<0.0001** | **<0.0001** | **<0.0001** |  |
| **DMSO vs PND (a)** | **0.0169** | **0.5457** | **<0.0001** | **0.0289** | **0.0155** | **0.2018** | **0.0050** | **0.0013** | **0.0793** | **0.0007** | **<0.0001** | **0.0020** | **<0.0001** | **<0.0001** | **<0.0001** | **<0.0001** |  |
| **DMSO vs Das+PND (a)** | **<0.0001** | **<0.0001** | **<0.0001** | **<0.0001** | **<0.0001** | **<0.0001** | **<0.0001** | **<0.0001** | **<0.0001** | **<0.0001** | **<0.0001** | **<0.0001** | **<0.0001** | **<0.0001** | **<0.0001** | **<0.0001** |  |
| **Das vs PND (b)** | **<0.0001** | **0.0235** | **0.1319** | **0.0003** | **<0.0001** | **0.0007** | **<0.0001** | **0.0001** | **0.0005** | **<0.0001** | **0.0013** | **<0.0001** | **<0.0001** | **0.0015** | **<0.0001** | **0.0026** |  |
| **Das vs Das+PND (b)** | **<0.0001** | **<0.0001** | **<0.0001** | **<0.0001** | **<0.0001** | **<0.0001** | **<0.0001** | **<0.0001** | **<0.0001** | **<0.0001** | **<0.0001** | **<0.0001** | **<0.0001** | **<0.0001** | **<0.0001** | **<0.0001** |  |
| **PND vs Das+PND (c)** | **<0.0001** | **<0.0001** | **<0.0001** | **<0.0001** | **<0.0001** | **<0.0001** | **<0.0001** | **<0.0001** | **<0.0001** | **<0.0001** | **<0.0001** | **<0.0001** | **<0.0001** | **<0.0001** | **<0.0001** | **<0.0001** |  |
